# Potential of Encapsulated Bovine Colostrum in Powder-Based Formulations for Facial Clay, Peel-Off Gel, and Sleeping Gel Masks

**DOI:** 10.3390/gels11020111

**Published:** 2025-02-04

**Authors:** Pornpansa Chuesomboon, Thomas Rades, Wantida Chaiyana

**Affiliations:** 1 Department of Pharmaceutical Sciences, Faculty of Pharmacy, Chiang Mai University, Chiang Mai 50200, Thailand; pornpansa_c@cmu.ac.th; 2 Department of Pharmacy, Faculty of Health and Medical Sciences, University of Copenhagen, Universitetsparken 2, 2100 Copenhagen, Denmark; thomas.rades@sund.ku.dk; 3 Center of Excellence in Pharmaceutical Nanotechnology, Faculty of Pharmacy, Chiang Mai University, Chiang Mai 50200, Thailand; 4 Multidisciplinary and Interdisciplinary School, Chiang Mai University, Chiang Mai 50200, Thailand

**Keywords:** encapsulation, bovine colostrum, nutritional, coacervation, freeze-dry, conventional clay mask, peel-off gel mask, sleeping gel mask

## Abstract

Bovine colostrum is a bioactive compound with potential in cosmetic applications but has a limited shelf life. This study aimed to develop an effective encapsulation system for bovine colostrum using the complex coacervation method and incorporate it into powder formulations for facial masks. The research explored various gelatin-to-gum Arabic ratios to optimize the physical and chemical stability, encapsulation efficiency, and loading capacity of the encapsulated bovine colostrum (EBC). The EBC was further incorporated into powder formulations for clay masks, peel-off gel masks, and sleeping gel masks. The optimal gelatin-to-gum Arabic ratio was found to be 2:1, yielding the highest entrapment efficiency (66.6 ± 3.3% *w*/*w*) and loading capacity (67.6 ± 3.4% *w*/*w*) of bovine colostrum. For clay masks, the most effective powder blend incorporating EBC enhanced the moisture content, water solubility, and hygroscopicity, without affecting the drying time (9.7 ± 0.6 min). Additionally, peel-off gel masks incorporating EBC significantly reduced water activity and improved moisture content and hygroscopicity, while the drying time decreased from 44.3 ± 0.6 to 25.0 ± 1.7 min. For sleeping gel masks, the formulation with EBC increased water activity, while other parameters remained stable. In conclusion, the EBC with enhanced stability was effectively integrated into various powders for facial mask formulations.

## 1. Introduction

Global dairy production and consumption have increased significantly over the decades, with major producers including the United States, Brazil, Argentina, India, and China [1]. According to the Department of Livestock Development in Thailand, there are approximately 740,729 dairy cows in the country, producing about 2821.86 tons of raw milk daily (2023). Colostrum, a biofluid produced in the initial days postpartum, accounts for only about 0.5% of a cow’s annual milk output [2]. However, bovine colostrum remains largely underutilized, as most healthy dairy cows produce it in quantities that far exceed the needs of their calves [2].

Bovine colostrum is critical for the calf’s early development, providing essential immunoglobulins necessary for passive immunity, and is notably rich in proteins, vitamins, and minerals [3,4]. In recent years, there has been growing interest in using colostrum from various animals in cosmetics and dermatology [5]. The utilities of the bovine colostrum in the pharmaceutical and cosmetic area are listed in Table 1. The remarkable range of skin benefits associated with colostrum-based products include chronic wound healing, tissue regeneration, acne management, and sebum regulation [6]. Furthermore, sheep colostrum has been noted for its potential to reduce inflammation, improve skin hydration, provide protective and firming effects, and offer anti-aging and soothing properties [7]. Despite the promising benefits of bovine colostrum, its application faces several challenges. Its high water activity renders it susceptible to microbial contamination, including bacteria, yeasts, and molds [8]. Additionally, it is sensitive to heat, which can diminish its vitamin and mineral content [9] and its lipid components can oxidize, leading to rancidity [10]. These challenges highlighted the need for innovative approaches to fully leverage the potential of colostrum in diverse applications, including in the context of cosmetics.

Encapsulation to protect bioactive compounds from environmental factors like light, moisture, and oxygen [20] emerges as a sophisticated technique to address the challenges of incorporating colostrum into the formulations by creating a physical barrier around the bioactive components to shield them from unfavorable conditions. Various common encapsulation methods, including spray-drying, freeze-drying, fluid bed coating, melt extrusion, emulsification, electrospraying, and coacervation, may be used [21,22,23]. Among these methods, complex coacervation is a popular approach for microencapsulation, due to its simplicity and effectiveness [24]. Complex coacervation can be achieved in protein–polysaccharide biopolymer mixtures by precisely controlling external parameters, which facilitate electrostatic interactions between oppositely charged macromolecules [25]. Various protein and polysaccharide combinations have been reported for the development of complex coacervation, including gelatin with gum Arabic [26], soybean protein isolates with pectin [27], whey protein with gum Arabic [28], canola protein isolate with chitosan [29], and gelatin with acacia gum [30], as well as gelatin with pectin [31]. Among these, the gelatin and gum Arabic system is one of the earliest and most extensively studied [26].

For cosmetic purposes, encapsulated bovine colostrum (EBC) can be integrated into powder formulations for facial masks, preserving its beneficial properties. Such masks can be formulated in various forms, including traditional clay masks that require rinsing, sleeping gel masks that are left on overnight, or peel-off masks that are removed after drying, depending on the specific additives and formulation goals. Therefore, the aims of the present study were to develop an encapsulation system for bovine colostrum and integrate it into powder formulations for various types of facial masks. By optimizing the encapsulation process, we aimed to improve the stability of bovine colostrum in cosmetic applications, potentially transforming its use in skincare products.

## 2. Results and Discussion

### 2.1. Bovine Colostrum and Its Chemical Compositions

Bovine colostrum is a yellowish milky liquid with a slightly acidic pH of 6.4. The nutritional composition of bovine colostrum is shown in Table 2. Fat was found to be present at 4.80 ± 0.99% *w*/*w*, while solids-not-fat were found to be present at 13.14 ± 0.22% *w*/*w*, composed mainly of protein, along with lactose and trace amounts of minerals and vitamins. The findings were consistent with a previous study that reported bovine colostrum contains a high protein content (~15% *w*/*w*) relative to fat (4 to 6% *w*/*w*) and lactose (3 to 4% *w*/*w*) [32]. However, the previously reported protein content exhibited considerable variability, ranging from 4.1% to 14.0% *w*/*w*, while the fat concentration remained within a narrower range of 3.9% to 6.7% *w*/*w* [6]. Additionally, Puppel et al. (2019) reported that protein was a major constituent of bovine colostrum (14.56% *w*/*w*), which was obviously higher than those found in milk (3.3% *w*/*w*) [9]. Similarly, Mehra et al. (2021) reported that only 3.7% to 4.0% *w*/*w* of protein was detected in milk, whereas bovine colostrum exhibited protein levels between 4.1% and 14.0% *w*/*w* [6]. Regarding the literature, significant variations in the nutrient ranges, particularly in the amounts of fat and protein, of bovine colostrum have been observed, influenced by factors such as breed, genetic factors, season, temperature stress, dry periods, nutrition, and metabolic disturbances [6]. Overall, however, these nutritional components make bovine colostrum beneficial not only for dietary purposes but also for cosmetic applications.

In the context of topical applications, bovine colostrum holds significant biological potential, with its natural and non-toxic properties making it highly valuable in cosmetics and dermatology [5]. Fat and protein, which are typically more abundant in bovine colostrum than in milk, play essential roles in supporting energy provision and muscle development in newborn calves [9]. In addition, the protein levels and lipid compositions in bovine colostrum are also vital for its therapeutic efficacy, particularly in skin care and human health [33]. In cosmetic formulations, these components are valued for their moisturizing and nourishing properties [34]. In addition to its nutritional components, bovine colostrum is rich in immune factors, growth factors, and tissue repair factors [35]. Bovine colostrum has been reported to enhance skin fibroblast proliferation, promote wound healing, and reduce inflammation [5,36]. Its topical formulations support recovery from burn wounds, ulcers, and sunburns, as well as showing potential in treating conditions such as acne vulgaris, plaque psoriasis, and contact lesions [5]. In addition, bovine colostrum preparations have been reported to significantly enhance the healing of difficult-to-treat wounds, including those caused by buttock erythematosus and erosion erythema [37]. Additionally, cosmetic products featuring bovine or equine colostrum, along with hyaluronic acid or its salts and olive oil or vitamin E, have demonstrated benefits in enhancing skin elasticity and firmness, offering moisturizing and antioxidant effects, as well as diminishing skin sagging and age spots in the healthy skin of elderly individuals [38].

Although bovine colostrum has been known for its rich nutritional properties and a variety of benefits, it has a relatively short shelf life, typically spoiling within a few days, even in a refrigerator [39]. Therefore, various methods have been employed to prevent spoilage and the growth of pathogenic microorganisms in bovine colostrum, thereby extending its shelf life, including the addition of chemical preservatives, heat treatment (such as pasteurization), refrigeration or cooling, freezing, freeze-drying, and spray-drying [40]. In the current study, a combination of several methods was used to preserve the bovine colostrum. Following the collection of bovine colostrum, it was initially frozen for transportation from the farm to the laboratory. Upon arrival, the bovine colostrum was thawed, pasteurized at 70 °C for 2 min, and then subjected to freeze-drying. The rationale behind these steps stems from the fact that while freezing prevents the growth and multiplication of microorganisms, their spores can survive and potentially grow upon the thawing of bovine colostrum [41]. Therefore, an additional pasteurization following the thawing process was conducted to eliminate objectionable organisms while minimizing chemical alterations [42]. However, pasteurized bovine colostrum can typically be stored without refrigeration for only 2–3 months [40], prompting the use of freeze-drying, which is particularly effective at removing moisture while preserving nutrient integrity [43]. In addition to significantly extending shelf life through the freeze-drying process, it also enhanced convenience for long-term storage and consumption [43].

The amino acid composition of freeze-dried bovine colostrum, as shown in Table 3, indicates that a significant amount of various amino acids can be detected, with glutamic acid being the most abundant, followed by leucine and proline. The results were consistent with previous reports indicating that glutamic acid is the most abundant amino acid in bovine colostrum, while variations were found in other amino acids with lower amounts [44]. Although glutamic acid is a nonessential amino acid since it can be synthesized in the human body, nine amino acids (histidine, isoleucine, leucine, lysine, methionine, threonine, phenylalanine, tryptophan, and valine), which are essential, were detected in the freeze-dried bovine colostrum. However, the total and individual amino acids were present in lower concentrations in the present study compared to previous reports, which revealed that bovine colostrum contained 14–16% *w*/*w* protein content [36] and 99.92 mg of glutamic acid per g protein, accounting for 9.99% *w*/*w* [45]. This could be due to various factors including different breeds of cow and the degradation of individual amino acids via deamidation and dephosphorylation [46].

### 2.2. Encapsulated Bovine Colostrum (EBC)

**Figure 1:**
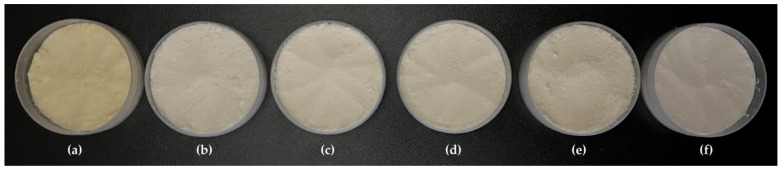
Physical appearance of the freeze-dried bovine colostrum (**a**) and encapsulated bovine colostrum (EBC) prepared by complex coacervation using various ratios of gelatin and gum Arabic, including 1:0 (**b**), 2:1 (**c**), 1:1 (**d**), 1:2 (**e**), and 0:1 (**f**).

Bovine colostrum was successfully encapsulated using gelatin and gum Arabic. The physical appearance of each EBC is shown in Figure 1. The freeze-dried bovine colostrum was a fine powder with a pale-yellow color, while all EBC exhibited an almost white color, which was notably paler compared to the native freeze-dried bovine colostrum. The findings were in line with the data obtained from a colorimetric test as shown in Table 4. The encapsulation in gelatin and gum Arabic led to a decrease in both the a* and b* values. The a* axis is relative to the green–magenta opponent colors, with negative values indicating a shift towards green and positive values indicating a shift towards magenta, while the b* axis represents the blue–yellow opponents, with negative values indicating a shift towards blue and positive values indicating a shift toward yellow. Therefore, decreases in the a* and b* values signify a shift in color perception, with a reduction in the a* value implying a movement away from the magenta tint toward the green hue and a reduction in the b* value indicating a transition from the yellow tone toward the blue shade. Although the lightness (L*) of all formulations was comparable, the freeze-dried bovine colostrum exhibited the most yellowish hue, with a b* value of 18.29 ± 0.23 (*p* < 0.05). The yellow hue of bovine colostrum was about double that of the ECB, with the b* value ranging from 9.02 ± 0.24 to 10.92 ± 0.11. It was likely that after encapsulation, the yellow color of the bovine colostrum was masked by the polymer, particularly in formulations containing a higher amount of gum Arabic, with the significantly lowest b* value of 9.02 ± 0.24 (*p* < 0.05). On the other hand, although a statistically significant difference was observed in the a* values, the color hues of green or red were not detectable in either the native freeze-dried bovine colostrum or its encapsulations. The observed results can be attributed to the low a* values of each formulation, which ranged from −0.01 ± 0.05 to 1.09 ± 0.09.

**Figure 2:**
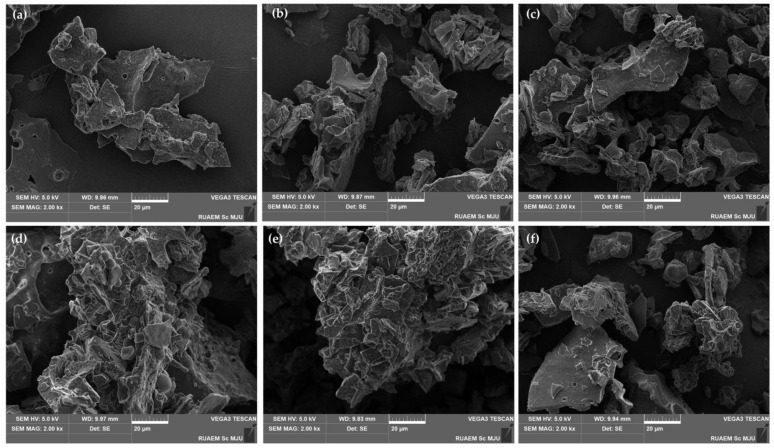
Scanning electron microscopic (SEM) images at a magnification of 2.00 k×, with a scale bar indicating 20 μm, of the freeze-dried bovine colostrum (**a**) and encapsulated bovine colostrum (EBC) prepared by complex coacervation using various ratios of gelatine and gum Arabic, including 1:0 (**b**), 2:1 (**c**), 1:1 (**d**), 1:2 (**e**), and 0:1 (**f**).

In addition to the visual examination of the external appearance of the freeze-dried bovine colostrum and its encapsulations, scanning electron microscopic (SEM) images at a magnification of 2.00 k× are presented in Figure 2. The freeze-dried bovine colostrum exhibited an irregular, sawdust-like morphology with a smooth or slightly rough surface texture. The results were consistent with a previous study, which noted that the dried milk powder produced by freeze-drying displayed irregular structures, whereas the powder from spray-drying was characterized by spherical particles, often featuring wrinkled or folded surfaces [47]. In addition, the encapsulation with native gelatin or gum Arabic exhibited a similar morphology of irregular shapes with smooth surfaces. This can likely be attributed to the hydrophilic nature of both gelatin and gum Arabic [48], which affects their interaction with the core material and influences the surface characteristics of the encapsulated particles, potentially leading to smoother surfaces. Additionally, their ability to form films or gels allowed both materials to effectively encapsulate the bovine colostrum by creating a protective barrier around the core material. Gum Arabic has been previously reported for its excellent water-holding properties, which contribute to the formation of a smooth surface on frozen items by inhibiting the growth of ice crystals [49].

Aside from external appearance and morphology, the freeze-dried bovine colostrum and its encapsulation were evaluated for yields, water activity, moisture content, water solubility, hygroscopicity, and protein content, as shown in Table 5. The yield of freeze-dried bovine colostrum was found to be 14.16 ± 0.21% *w*/*w*, which was primarily attributed to the loss of water present in the colostrum prior to freeze-drying. Therefore, the freeze-drying process applied to bovine colostrum effectively demonstrates its capability to remove water while concentrating the critical components of the colostrum. It was observed that the encapsulations had a higher weight due to the presence of the additional coating polymers. Although a significant difference was not detected (*p* > 0.05), the trend in the changing yield could be observed. Increasing the proportion of gelatin in the mixture generally led to an increase in yield. This suggested that gelatin plays a crucial role in the process, improving the encapsulation efficiency. Additionally, gum Arabic also contributed to the yield, but its effect appeared to be less pronounced than that of gelatin. A combination of both components was found to optimize the yield. The likely explanation is that gelatin provides a highly homogeneous blend with excellent film-forming properties and good mechanical strength [50], while gum Arabic may contribute to adhesive and binding capabilities [51]. The interaction between gelatin and gum Arabic might create crosslinks that improve the stability and structure of the final encapsulation product, resulting in a higher yield. However, gelatin not only yielded a film exhibiting good mechanical properties but also provided excellent water solubility [50]. Additionally, gelatin is recognized for its ability to act as a carrier for hydrophobic compounds, thereby improving their solubility in aqueous media [52]. Likewise, gum Arabic is known to possess excellent water solubility and surface-active properties [53]. Therefore, the encapsulations demonstrated significantly improved water solubility (*p* < 0.05), as shown in Table 5. This water solubility enhancement suggested that the encapsulation process effectively increased the ability of the encapsulated materials to dissolve in water, which was attributed to the properties of the encapsulating agents or coating polymers. On the other hand, water activity, which is a desirable parameter in encapsulated products as it contributes to longer shelf life, stability, and preservation of the product’s quality, was found to be significantly reduced in the EBC (*p* < 0.05). Therefore, it can be concluded that encapsulation is effective in improving the water solubility of bovine colostrum while simultaneously decreasing its water activity, thereby potentially enhancing its stability and quality. However, the moisture content and hygroscopicity of the EBC were not significantly different from those of native bovine colostrum.

### 2.3. Stability of EBC

**Figure 3:**
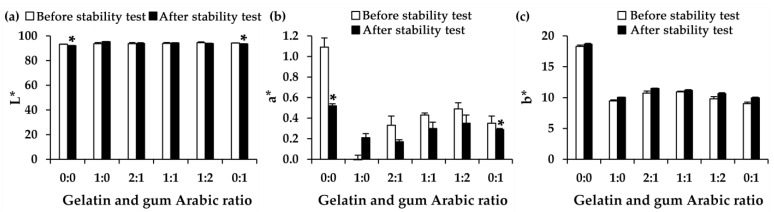
The color in terms of L* (**a**), a* (**b**), and b* (**c**) of the freeze-dried bovine colostrum and encapsulated bovine colostrum (EBC), prepared by complex coacervation using various ratios of gelatin and gum Arabic. The asterisk (*) indicates a statistically significant difference between the color parameter before (white bar) and after (black bar) eight cycles of heating and cooling conditions, as determined by pairwise t-tests (*p* < 0.05).

Bovine colostrum is rich in essential nutrients, including bioactive proteins, vitamins, minerals, growth factors, and both free and conjugated oligosaccharides [15,54]. However, the application of these bioactive components is challenged by their physical and chemical instability during processing and storage. Maintaining the stability of these bioactive components, particularly proteins, is crucial to preserving the beneficial effects of bovine colostrum. The stability of the freeze-dried bovine colostrum, along with its encapsulations, was evaluated after being exposed to repetitive temperature fluctuations through eight cycles of heating (45 °C) and cooling (4 °C) conditions. Although these conditions do not simulate real-world storage scenarios, they are well-known accelerated conditions for stability testing [55]. The repeated heating/cooling cycles of the formulations have been widely used to ensure the stability, reliability, and possible reversibility of the microencapsulations [56]. The physical appearance of both the original freeze-dried bovine colostrum and its encapsulated product did not change after being subjected to stability testing. However, colorimetric analysis using a colorimeter revealed subtle alterations in color that were not perceptible through visual inspection, as shown in Figure 3.

Bovine colostrum exhibited significant alterations in the L* and a* values during the stability study through eight cycles of heating and cooling conditions. The significant reduction in the L* value indicated a darker color, while the notable decrease in the a* value, from 1.09 ± 0.09 to 0.52 ± 0.02, signified a fading of the red hue (*p* < 0.05). On the other hand, the EBC remained consistent in color with its initial state, particularly in the formulation that utilized a combination of gelatin and gum Arabic. The findings were in line with a previous study that noted that freeze-drying proved a suitable method for the color stabilizing of natural extracts [57]. However, encapsulation solely with gum Arabic resulted in significant alterations in all color parameters (L*, a*, and b*). A likely explanation could be the inherent color of gum Arabic, which ranges from very pale yellowish-brown to dark reddish-brown, depending on the tannin content in the sample [58], which could fade over the stability test. Nonetheless, gum Arabic has been previously reported to be a color preservative to stabilize natural extracts, e.g., saffron and beetroot extracts [57].

**Figure 4:**
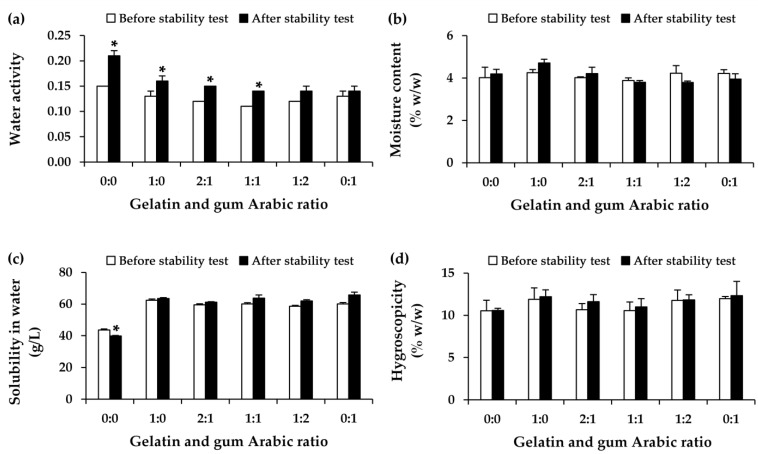
Water activity (**a**), moisture content (**b**), solubility in water (**c**), and hygroscopicity (**d**) of the freeze-dried bovine colostrum and the encapsulated bovine colostrum (EBC), prepared by complex coacervation using various ratios of gelatin and gum Arabic. The asterisk (*) indicates a statistically significant difference between the color parameter before (white bar) and after (black bar) eight cycles of heating and cooling conditions, as determined by pairwise t-tests (*p* < 0.05).

Additionally, the water activity and water solubility of the native bovine colostrum experienced notable changes following the stability test (*p* < 0.05), as shown in Figure 4. Increasing water activity led to an accelerated growth rate of microbial contamination, as water is the primary factor influencing microbial spoilage in foods [59,60]. Therefore, the freeze-dried bovine colostrum was found to increase water activity, resulting in heightened susceptibility to microbial contamination. The encapsulations tended to maintain the stability of bovine colostrum with a smaller increase in water activity after the stability test, and this effect was most notably observed at a higher proportion of gelatin. Therefore, the encapsulation technique is recommended for maintaining the stability of bovine colostrum with respect to water activity, particularly when using gum Arabic as a wall material. Although the encapsulation effectively maintained water activity compared to native bovine colostrum, the system with gelatin, particularly at higher gelatin ratios, showed a significant increase in water activity. This can be explained by the moisture-sensitive properties of gelatin [61]. Therefore, integrating gelatin with an additional wall material, e.g., gum Arabic, would offset the limitations of each component, leading to a more effective encapsulation system. However, all EBC (which exhibited increased water solubility compared to the native bovine colostrum) maintained this solubility throughout the stability study, whereas native bovine colostrum showed a significant reduction in water solubility. Hence, it can be concluded that EBC demonstrated a greater potential for maintaining stability compared to native bovine colostrum. The EBC was preserved in its initial state regarding moisture content, water solubility, and hygroscopicity.

**Figure 5:**
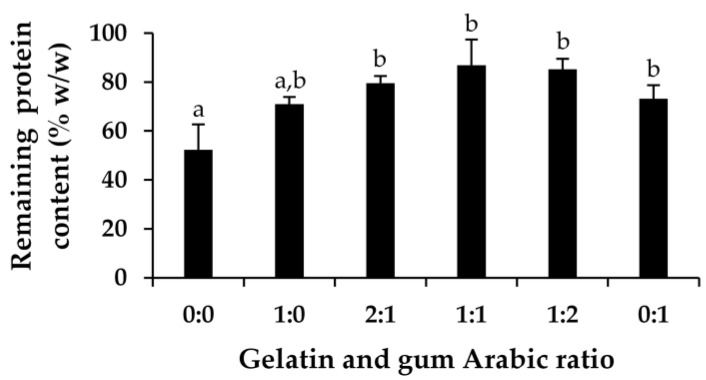
Remaining total protein content of the freeze-dried bovine colostrum and the encapsulated bovine colostrum (EBC), prepared by complex coacervation using various ratios of gelatin and gum Arabic. Lowercase letters (a and b) in each column indicate statistically significant differences, as determined by one-way ANOVA followed by Tukey’s post hoc test (*p* < 0.05).

In addition to assessing physical stability, EBC has also been evaluated for its protein content, as protein is recognized as the primary component of bovine colostrum. The remaining total protein content was evaluated after being exposed to repetitive temperature fluctuations through eight cycles of heating and cooling. The findings, as illustrated in Figure 5, revealed that the total protein content of the freeze-dried bovine colostrum was reduced to nearly half after the stability test, decreasing to 52.4 ± 10.4%. In contrast, encapsulation has been effective in protecting the protein from degradation.

Gelatin alone showed a tendency to enhance stability, with the remaining total protein content detected at 70.9 ± 2.9%. Similarly, gum Arabic alone significantly improved stability, with a remaining total protein content of 73.2 ± 5.4% (*p* < 0.05). The combination of both polymers proved to be even more effective, with the remaining total protein content reaching 79.6 ± 2.8%, 86.8 ± 10.6%, and 85.2 ± 4.2% at gelatin-to-gum Arabic ratios of 2:1, 1:1, and 1:2, respectively. The likely explanation might be attributed to the complex formed between gelatin (positively charged) and gum Arabic (negatively charged), where polysaccharide–protein interactions are stabilized by electrostatic forces between the two polymers [62]. As proteins carry positive charges, they can interact with negatively charged polysaccharides [63]. Gum Arabic, a polysaccharide from acacia trees, is commonly paired with gelatin, a protein derived from collagen, since they form stable complexes through these electrostatic interactions [64]. Therefore, encapsulation could improve protein stability by shielding proteins from environmental stressors like heat, moisture, and oxygen, which can lead to degradation [65,66,67].

### 2.4. Entrapment Efficiency and Loading Capacity of EBC

**Figure 6:**
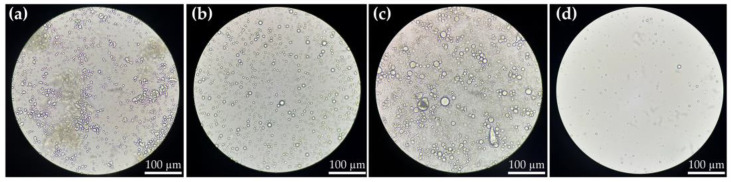
Light micrographs (400× magnification, with a scale bar indicating 100 μm) of bovine colostrum (**a**), the supernatant of bovine colostrum after centrifugation (**b**), encapsulated bovine colostrum (**c**), and the supernatant of encapsulated bovine colostrum after centrifugation (**d**) at 4000 rpm for 20 min.

**Figure 7:**
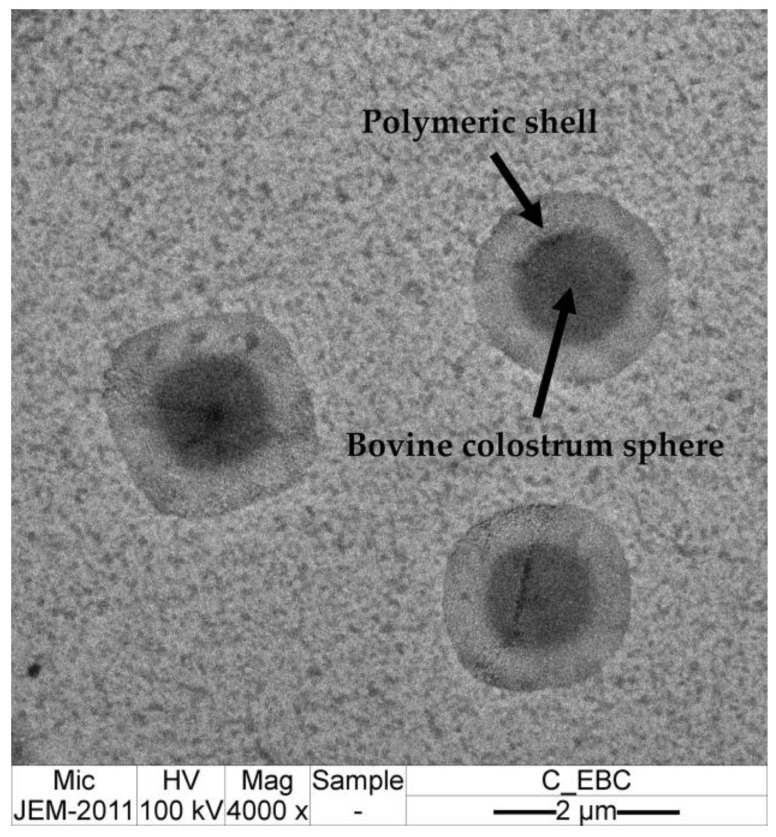
Transmission electron micrographs (4000× magnification, with a scale bar indicating 2 μm) of encapsulated bovine colostrum.

An indirect method, which measured the free bovine colostrum not encapsulated by evaluating the total protein content, was used to assess both entrapment efficiency, and loading capacity in the current study. To prevent a protein-free supernatant caused by the sedimentation of free protein after centrifugation, which could result in a false positive for encapsulation, preliminary studies were conducted. Both bovine colostrum and encapsulated bovine colostrum (EBC) were centrifuged, and their supernatants were characterized under a microscope in comparison to the native samples that had not undergone centrifugation. The micrographs shown in Figure 6 indicate that fat globules are present in the bovine colostrum and were not sedimented after centrifugation, as they remained detectable in the supernatant. These results were confirmed by the similar total protein content of bovine colostrum and its supernatant after centrifugation, as shown in Table 6. The findings suggest that the proteins did not sediment under the conditions used for the indirect entrapment efficiency determination method. Additionally, the total protein content was similar to that of EBC, indicating that the total protein measurement accurately represents the amount of bovine colostrum. In contrast, a distinctly lower protein content was detected in the supernatant of EBC after centrifugation, reduced by half from 9.8 ± 0.0 to 4.6 ± 0.1 mg/mL (*p* < 0.05), confirming that no false positive for encapsulation occurred. Additionally, the transmission electron microscopy (TEM) micrographs of EBC, shown in Figure 7, reveal the morphology and structural characteristics of the encapsulated particles, further supporting the efficiency and integrity of the encapsulation process. A core–shell structure was observed, characterized by distinct polymeric shells surrounding the bovine colostrum spheres. The wall thickness was measured to be around 400 and 500 nm, providing strong evidence for successful encapsulation within the microcapsules.

The entrapment efficiency and loading capacity of bovine colostrum in the encapsulated systems are shown in Table 7. As encapsulation efficiency signifies the effectiveness of a process in enclosing a substance within a carrier system, a higher encapsulation efficiency suggests that a greater portion of the substance has been successfully enclosed within the carrier. The findings indicate that using gelatin alone resulted in significantly higher entrapment efficiency and loading capacity compared to using gum Arabic alone or the combination of both coating materials (*p* < 0.05). Therefore, gelatin was found to have a strong effect on the loading of bovine colostrum in the encapsulation system. Statistically, however, the combination of gelatin and gum Arabic at a 2:1 ratio exhibited equivalent entrapment efficiency and loading capacity to that of the encapsulation system using gelatin alone. Therefore, the mixture of gelatin and gum Arabic would be preferable, as previous findings suggested it significantly enhanced the chemical stability of bovine colostrum. The findings were in line with the previous study by Marfil et al. (2018), which reported that the highest values of entrapment efficiency occurred at an equal ratio of gelatin to gum Arabic (1:1), as well as at a 2:1 ratio, regardless of the concentration of wall materials [68]. Lv et al. (2013) also recommended a 2:1 gelatin-to-gum Arabic ratio, as it enables the smaller gelatin molecules to effectively extend and interact with the larger gum Arabic molecules through charge neutralization [69]. At this ratio, gelatin molecules transitioned from a flexible state to an ordered polyproline-II (PPII) helix, while gum Arabic molecules shifted from a partly ordered PPII helix to a compact polyproline-I (PPI) helix [69]. These molecular transformations of both polymers ultimately contributed to the formation of smaller coacervates, which are more favorable for applications requiring controlled size and stability [69].

Gelatin, a biodegradable protein derived from collagen, is a first commercial choice as wall material due to its excellent film-forming properties, emulsifying capacity, thickening ability, water solubility, high stabilizing activity, and high crosslinking activity through its primary amino group [53]. Gelatin has been used as a coating or wall material for the encapsulation of various natural extracts. Encapsulation of curcumin in gelatin microspheres significantly improved both curcumin’s water solubility and bioavailability by 38.6- and 11.3-fold higher than commercial curcumin, respectively [52]. In addition to the hydrophobic compounds, gelatin capsules were capable of stabilizing a water-soluble (−)-epigallocatechin gallate against degradation in an aqueous solution [70]. However, a single encapsulating matrix is not supposed to possess all the required characteristics, and efforts to improve encapsulation properties have been made by using mixtures of carbohydrates with proteins and polysaccharides in different proportions [53]. Gum Arabic, a highly branched polymer with units of galactose, rhamnose, arabinose, and glucuronic acid [53], has been widely used along with gelatin to form the complex coacervation. In line with this, the results of the current study suggest that the encapsulation system composed of a 2:1 ratio of gelatin to gum Arabic is the most suitable, due to its highest entrapment efficiency and loading capacity, as well as its good physical and chemical stability-enhancing properties.

The EBC developed in the current study incorporates gelatin and gum Arabic, both of which are biodegradable polymers. Gelatin, an animal-derived protein, consists of 19 amino acids linked by peptide bonds, which are hydrolyzed by proteolytic enzymes into individual amino acids [71]. In addition, gum Arabic is a polysaccharide characterized by a highly branched arabinogalactan structure, and it is both biocompatible and biodegradable [72]. Thus, this aligns with the concept of sustainable cosmetics, as both polymers come from natural, renewable sources and are capable of naturally degrading in the environment, helping to reduce long-term waste. Additionally, the biodegradability of these polymers ensures that they do not persist in the environment, supporting eco-friendly product formulations that contribute to reducing the overall ecological footprint of cosmetic products. Aside from the biodegradability and sustainability of the developed EBC, these natural polymers are readily available at a reasonable cost, resulting in an affordable production process. Furthermore, the simplicity of the production process enables efficient large-scale manufacturing, and the developed encapsulation systems do not require exhaustive purification processes [71].

### 2.5. Blank Powders for Facial Masks

A blank powder for facial masks was developed to be freshly mixed with water before application, which is an approach to maintaining the freshness and potency of the active ingredients. Various types of blank powder for facial masks were developed in the current study, including conventional clay masks, peel-off gel masks, and sleeping gel masks. The powder for a conventional facial clay mask typically forms a paste after mixing with water and can be applied directly to the face for a short period before being washed off. On the other hand, the powder for a facial peel-off gel mask forms a gel after mixing with water, which can be applied directly to the face. After being left to dry, the gel turns into a film that can be peeled off. In contrast, the powder for a sleeping gel mask forms a gel after mixing with water, which can be applied directly to the face and left on overnight.

#### 2.5.1. Blank Powder for Conventional Facial Clay Masks

Clays are cosmetically active ingredients known for their cleansing properties, as well as their anti-aging, anti-wrinkling, and sun protection benefits [73]. Talc and kaolin are among the most commonly used clay minerals in cosmetic products, especially in clay masks [73]. Therefore, the formulation of the blank powder for a conventional facial clay mask in the current study primarily consisted of talc and kaolin in different ratios (40:45, 30:55, and 20:65), which functioned as absorbents to remove excess oil and impurities from the skin [74]. In addition, magnesium aluminum silicate, a negatively charged clay with silicate layers, was incorporated into the formulation to function as an adsorbent, opacifier, and viscosity-increasing agent [75]. Titanium dioxide was incorporated to enhance the mask’s opacity, contributing to its aesthetic appeal of white color [76]. Silica served as a texturizing and absorbent agent, while allantoin was included for its skin-soothing and conditioning properties [77]. Finally, imidazolidinyl urea was used as a preservative, ensuring the stability and safety of the formulation by inhibiting microbial growth. The external appearance and characteristics of blank powders for conventional facial clay masks are shown in Table 8. All the formulations were found to be similar, as they all appeared as fine white powders. In addition, the lightness (L*) was found to be the same, while the a* and b* values significantly decreased at higher concentrations of kaolin (*p* < 0.05). Although significant differences were detected in some color parameters, with values ranging from 0.14 ± 0.02 to 0.35 ± 0.08 for a* and 3.48 ± 0.19 to 4.27 ± 0.30 for b*, these differences were too subtle to be observed visually. Formulation C3 had the most desirable characteristics, as it exhibited the significantly lowest water activity and the significantly highest water solubility. Since water activity is a crucial parameter in self-preservation and plays a significant role in controlling the survival of microorganisms in cosmetic formulations [78], the low water activity of C3 makes it an effective barrier to microbial growth and contributes to a promising extended shelf life. Therefore, C3 was selected for further mixing with water at various ratios to find out the optimum powder-to-water ratio for facial masks.

The results shown in Table 9 indicate that the blank powder for the conventional facial clay mask mixed with water at ratios of 1:1, 1:2, and 1:3 generated a white paste with a pH of 6.75 ± 0.01, regardless of the amount of water added. Since the pH of the skin is around 5.5, a pH range of 5–7 is considered acceptable for topical formulations to avoid skin irritation [79]. Regarding drying time, which plays a crucial role in both product performance and consumer perception, careful optimization is essential. An optimum drying time enables the mask to effectively perform its purifying and detoxifying functions while ensuring user comfort [80]. Insufficient drying time may compromise the mask’s ability to draw out impurities, while excessive drying time can cause discomfort and tightness, potentially discouraging regular use [80]. The current study determined the optimal amount of water to add to the blank powder for the conventional facial clay mask to achieve an optimum drying time. It was found that the amount of water added influenced drying time, with higher water content leading to longer drying times. Therefore, the 1:1 ratio, which dried in the shortest time of 7.67 ± 0.58 min, was selected for further incorporation of the EBC, as it offers the most convenience and is most favorable for consumer perception.

#### 2.5.2. Blank Powder for Facial Peel-Off Gel Mask

A peel-off gel mask is a type of skincare product that is gently applied to the facial skin and removed after a few minutes [81]. It is commonly used to address facial skin issues like wrinkles, aging, and acne, and is particularly effective for unclogging pores that have become blocked by dust [81]. In the present study, a powder formulation for a facial peel-off gel mask was developed to incorporate EBC, thereby preserving the active compounds in a solid state. The formulation is designed to be mixed with water to form a gel prior to application on the skin. Upon the evaporation of water from the applied formulation, a dry film is formed, which can then be peeled off. Therefore, the formulation of the blank powder for the facial peel-off gel mask mainly consists of sodium alginate, which acts as the gelling agent to create a smooth, uniform gel, and calcium sulfate, which functions as a setting agent when combined with sodium alginate to allow the mask to set and form a solid film that can be peeled off [82]. The additives were also incorporated into the formulation to enhance the aesthetic properties of the peel-off gel mask. Tetrasodium pyrophosphate, frequently utilized in food and known for minimizing whey separation issues in dairy products, was employed as a stabilizer [83]. In addition, it also served as a buffering agent to control the pH of the formulation after mixing with water. On the other hand, diatomaceous earth and talc were incorporated into the formulation for their absorption properties, targeting dirt and facial sebum [84,85]. Different weight ratios of diatomaceous earth to talc (55:22, 65:12, and 75:2) were used in the formulations of blank powder for facial peel-off gel masks. Sodium metabisulfite and sodium sulfite were used as antioxidants to prevent ingredient oxidation, which could otherwise lead to product degradation, discoloration, and reduced efficacy. Additionally, both sodium metabisulfite and sodium sulfite possess antimicrobial properties, with sodium sulfite being particularly noted for its antifungal effects in powder formulations [86].

The external appearance and characteristics of blank powder for facial peel-off gel masks are shown in Table 10. All the formulations appeared as light-yellow powders, with the color becoming more intense as the amount of talc decreased. All color parameters correlated well with visual inspection. With lower concentrations of talc, L* significantly decreased, while both a* and b* significantly increased (*p* < 0.05). On the other hand, the water activity and hygroscopicity of each formulation were similar. However, formulation P3 had the lowest moisture content and was the most water-soluble (*p* < 0.05). Therefore, P3 was selected for further testing with water at various ratios to determine the optimal powder-to-water ratio for the facial mask.

The results shown in Table 11 indicate that the blank powder for the facial peel-off gel mask, when mixed with water at ratios of 1:1, 1:2, 1:3, and 1:4, produced distinctly different preparations. The 1:1 powder-to-water ratio was insufficient to fully wet the powder. In contrast, a 1:2 ratio produced an opaque paste, while a 1:3 ratio resulted in a viscous gel, and a 1:4 ratio yielded a viscous liquid. After mixing with water, the pH of the preparations ranged from 6.00 to 6.50. The drying time increased with a higher proportion of water added. However, while the pH and drying time of formulations with powder-to-water ratios of 1:3 and 1:4 were similar, the appearance of the 1:3 ratio as a viscous gel was more suitable for application compared to the 1:4 ratio, which was a viscous liquid. Therefore, the 1:3 ratio was considered the most suitable, as it provided a gel texture ideal for a peel-off gel mask.

#### 2.5.3. Blank Powders for Facial Sleeping Gel Masks

Facial sleeping gel masks, available in gel or cream forms, are applied to the face and left on overnight to deliver more moisture and emollience compared to regular masks. This study is the first to develop a powder formulation for a facial sleeping gel mask incorporating EBC to preserve active compounds in a solid state. The primary ingredient in the formulation was a gelling agent. The copolymer HK-AVC03 (ammonium acryloyldimethyltaurate/vinylpyrrolidone copolymer), a popular choice for gelling agents in various industries, including personal care, cosmetics, and pharmaceuticals, was used as a gelling agent in the current study. HK-AVC03 concentrations were varied within the range of 20% *w*/*w* to 60% *w*/*w* to determine the optimum level. Additionally, some active ingredients were also added to the formulation in the dried powder form, including niacinamide, allantoin, *Aloe vera* powder, and sodium hyaluronate. Niacinamide and allantoin are recognized for their skin-whitening and soothing effects, respectively, whereas *Aloe vera* powder and sodium hyaluronate are widely regarded for their moisturizing properties [87,88]. Disodium ethylenediamine tetra-acetic acid (disodium EDTA) was used to chelate metal ions, preventing them from catalyzing unwanted reactions that could lead to the degradation of the formulation. Additionally, imidazolidinyl urea was employed as a preservative to maintain the stability of the formulation by inhibiting microbial growth [89].

The external appearance and characteristics of the blank powder for sleeping gel masks are shown in Table 12. All the formulations were found to be similar, as they all appeared as white powders. All color parameters were found to be significantly different between S1 and S3 (*p* < 0.05), whereas S2 did not show a significant difference compared to any of the other formulations. However, the differences were too subtle to be observed visually. For instance, L* values ranged from 96.15 ± 0.37 to 96.76 ± 0.24, a* values ranged from 0.44 ± 0.06 to 0.59 ± 0.10, and b* values ranged from 3.26 ± 0.04 to 3.63 ± 0.19. On the other hand, with increasing concentrations of HK-AVC03, water activity significantly decreased while moisture content increased. In contrast, the hygroscopicity was found to be unchanged. Water solubility could not be determined as the gel readily formed upon the addition of water. Overall, formulation S2 had the most desirable characteristics, as it exhibited the significantly lowest water activity and moisture content. Therefore, S2 was selected for further mixing with water at various ratios to find the optimum powder-to-water ratio for facial masks.

The results shown in Table 13 indicate that mixing the powder with water at ratios of 1:5, 1:10, and 1:15 produced an opaque white gel with varying viscosity depending on the amount of water added. The 1:5 ratio resulted in a gel that was too viscous for application, while the 1:10 and 1:15 ratios were found to be suitable for forming the sleeping gel mask. However, the 1:10 ratio was selected for the further incorporation of the EBC, as it required less water and maintained a higher final concentration of all ingredients.

### 2.6. Powders for Facial Masks Containing EBC

The powder for facial masks, including conventional clay masks, peel-off gel masks, and sleeping gel masks, was successfully developed. The most suitable powder for each type of facial mask was selected for the incorporation of EBC. Overall, 50% of the EBC could be incorporated into the powder for conventional clay masks and sleeping gel masks, while only 20% *w*/*w* could be incorporated into the powder for peel-off gel masks. This was likely due to the high content of EBC interfering with the network formation of the gelling agent polymer chains, resulting in the failure to form a gel upon the addition of water.

The physical appearance and characteristics of both the blank formulations and those containing EBC are shown in Table 14. The external appearance of C3 and S2 was found to be similar, whereas formulation P3 exhibited a lower color intensity, despite the addition of only 20% *w*/*w* of EBC. However, color parameter measurements from the colorimeter revealed a significant increase in the b* value, indicating a more yellow color, in all formulations containing EBC compared to the blank formulations. On the other hand, the a* value was significantly increased in the conventional clay masks and peel-off gel masks, while it was significantly decreased in the sleeping gel masks. Although the color change is hardly detectable visually and is unlikely to impact consumer perception, it is important to note that it may be related to the quality of the product; therefore, it should be measured and recorded for quality control and product specification purposes.

Not only was the color of the formulation affected by the incorporation of EBC, but the water activity, moisture content, water solubility, and hygroscopicity were also altered. The EBC, with a moisture content of 4.02 ± 0.04% *w*/*w*, significantly impacted only the conventional clay masks, which initially had a low moisture content of 0.84 ± 0.11% *w*/*w*. In contrast, the peel-off gel masks and sleeping gel masks, which had a moisture content around 4% *w*/*w*, were not notably affected. The incorporation of EBC significantly enhanced the water solubility of the formulation, attributed to the high water solubility of the native EBC, which was as high as 59.46 ± 0.68 g/L. Similar to its effect on moisture content, the EBC, with a hygroscopicity of 10.66 ± 0.73% *w*/*w*, significantly increased the hygroscopicity of the conventional clay masks and peel-off gel masks, which initially had low hygroscopicity. However, it had no effect on the sleeping gel masks, which natively exhibited a high hygroscopicity. Hygroscopicity, or moisture sensitivity, refers to the tendency of solids (including powder cosmetic formulations) to absorb and retain water, which can lead to changes in physicochemical properties, formulation challenges, and shelf-life instability [90]. Therefore, formulations with lower hygroscopicity are more favorable.

The powder for the facial mask was characterized after being mixed with water at the appropriate ratios. Based on previous data, the suitable ratios were 1:1 for conventional clay masks, 1:3 for peel-off gel masks, and 1:10 for sleeping gel masks. It was found that the color of each formulation became more intensely yellow after mixing with EBC. These changes were visually observed after mixing with water, even though the formulations appeared the same as for the dry powder. The pH significantly decreased to around 6 after the incorporation of EBC, likely due to the acidic properties of the bovine colostrum. Interestingly, the addition of EBC to the powder for the peel-off gel mask significantly reduced the drying time to 25.00 ± 1.73 min, making it more suitable for application compared to the blank formulation.

## 3. Conclusions

In the present study, we successfully prepared EBC in a gelatin–gum Arabic matrix, resulting in a significant reduction in water activity and an enhancement of water solubility while maintaining the stability of the EBC. The optimal gelatin-to-gum Arabic ratio for encapsulation was identified, leading to improved entrapment efficiency and loading capacity. Three distinct powder formulations were developed for different types of facial masks, including conventional clay masks, peel-off gel masks, and sleeping gel masks. The EBC could be successfully incorporated into each of these mask formulations, offering potential benefits for skin health and rejuvenation. Further research, including in vivo or ex vivo tests and clinical trials, is needed to evaluate the efficacy of the formulation in enhancing skin hydration, anti-aging, and soothing effects, as well as the safety of these facial mask formulations.

## 4. Materials and Methods

### 4.1. Bovine Colostrum

The frozen bovine colostrum was purchased from local farmers at a dairy farm in San Kamphaeng District, Chiang Mai, Thailand. The colostrum from postpartum mother cows 1–4 days after calving that was left over after consumption by the calves and is typically discarded was collected by the farmers and immediately preserved through freezing. Therefore, as healthy cows produce colostrum in excess of the calf’s needs, the ethical considerations for the calves are not impacted [91,92]. Furthermore, the colostrum was not specifically collected for research purposes, but rather as a byproduct of normal farm management practices. Prior to the experiments, the frozen bovine colostrum was thawed to room temperature.

### 4.2. Chemical Materials

Food-grade gelatin from bovine skin was purchased from JR F&B Co., Ltd. (Bangkok, Thailand). Food-grade gum Arabic (*Acacia senegal*, 99.5% purity) was purchased from Krungthep Chemi Co., Ltd. (Bangkok, Thailand). Analytical-grade hydrogen chloride (HCl), sodium chloride (NaCl), and bicinchoninic acid (BCA) were purchased from Sigma Aldrich (St. Louis, MO, USA). Maltodextrin from maize starch (10–12% dextrose equivalent), calcium sulfate, sodium metabisulfite (99.3% purity), niacinamide (≥99.8% purity), Aloe vera (*Aloe barbadensis* leaf) gel spray-dried powder, sodium hyaluronate (≥95.0% purity), disodium ethylenediaminetetraacetic acid (EDTA), titanium dioxide (98.79% purity), talcum (60.04% *w*/*w* silica dioxide and 31.55% *w*/*w* magnesium oxide), and kaolin (47% *w*/*w* silica dioxide and 38% *w*/*w* aluminum oxide) were purchased at cosmetic grade from Krungthep Chemi Co., Ltd. (Bangkok, Thailand). Ammonium acryloyldimethyltaurate/vinylpyrrolidone copolymer (HK-AVC03) and allantoin USP (99.21% purity) were purchased at cosmetic grade from Cheme cosmetics (Bangkok, Thailand). Sodium sulfite, sodium alginate (ultrafine, 170 mesh), imidazolidinyl urea USP (99.19% purity), silica (MatteSilica 5™, 5 micron), magnesium aluminum silicate (ultra-hydrate, ClayThick™), and diatomaceous earth (3–5 micron) were purchased at cosmetic grade from Janjao Longevity Co., Ltd. (Bangkok, Thailand). Tetrasodium pyrophosphate (97.9% purity) was purchased at cosmetic grade from Hubei Xingfa Chemicals Group Co., Ltd. (Yichan, China).

### 4.3. Determination of Chemical Compositions of Bovine Colostrum by Milk Analyzer

The thawed bovine colostrum was investigated for the chemical compositions using an automatic milk analyzer (Dairy spec-combi, Bentley Instruments, Chaska, MN, USA). In brief, 20 mL of thawed bovine colostrum was pumped into a measuring cell and analyzed for the components of fat, solids-not-fat, protein, lactose, and total minerals. The analysis was performed with three replicates.

### 4.4. Freeze-Drying of Bovine Colostrum

The thawed bovine colostrum was pasteurized at a temperature of 70 °C for 2 min [27] and subjected to a freeze-drying process (EYELA FDU-2110, Tokyo Rikakikai Co., Ltd., Tokyo, Japan), carried out for a duration of 24 h at −70 °C, under a pressure ranging from 3 to 15 Pas. The resultant powder was stored in a laminate foil bag until further analysis at room temperature.

### 4.5. Determination of the Amino Acid Profile of Freeze-Dried Bovine Colostrum

The freeze-dried bovine colostrum powder was analyzed for its amino acid profile using an in-house method (TE-CH-372) based on the *Official Journal of the European Communities*, L257/16, employing the amino acid analyzer technique [28,29]. In brief, the sample with an injection volume of 20 µL was analyzed using a Biochrom 30+ Amino Acid Analyzer (Biochrom Ltd., Cambridge, UK), equipped with a column of oxidized high-performance sodium, a single flow cell with an optical beam splitter, and detection at wavelengths of 440 nm and 570 nm. The analysis duration was 1 h and 45 min.

### 4.6. Encapsulation of Bovine Colostrum by Complex Coacervation Method

#### 4.6.1. Preparation of Biopolymer Dispersion

Gelatin, gum Arabic, and their mixture were used as wall material polymers for the encapsulation of bovine colostrum since they are biopolymers from natural sources. Firstly, each individual biopolymer dispersion was prepared following the method of de Almeida Paula et al. (2019) [93]. The concentration of each biopolymer in the dispersion was 6% *w*/*w* [93,94]. In the case of gelatin, the biopolymer was first hydrated in distilled water for 30 min, followed by stirring at 200 rpm using an IKA C-MAG HS 10 magnetic stirrer (IKA-Werke GmbH & Co. KG, Staufen, Germany) at 50–60 °C for 25 min. The temperature was maintained not to exceed 60 °C to avoid hydrolysis of the gelatin. On the other hand, gum Arabic was dispersed in distilled water and stirred using an IKA C-MAG HS 10 magnetic stirrer (IKA-Werke GmbH & Co. KG, Staufen, Germany) set at 40 °C for 30 min [93].

#### 4.6.2. Complex Coacervation Between Gelatin and Gum Arabic

The complex coacervation of gelatin and gum Arabic was developed by gradually introducing a 6% *w*/*w* gelatin dispersion into a 6% *w*/*w* gum Arabic dispersion with continuous stirring at a constant temperature of 40 °C. The one-factor-at-a-time approach was used to systematically assess the impact of varying the gelatin-to-gum Arabic ratio on the formulation’s characteristics. Different ratios of gelatin and gum Arabic dispersions (1:0, 2:1, 1:1, 1:2, and 0:1) were prepared. Combining both biopolymers, the pH of the resulting mixtures was adjusted to 4.0 to promote complex coacervation by using a 1.0 N HCl aqueous solution. Subsequently, the mixtures were cooled down to 10 °C using an ice bath while stirring at 200 rpm using an IKA C-MAG HS 10 magnetic stirrer (IKA-Werke GmbH & Co. KG, Staufen, Germany) and stored at 7 °C for 2 h to minimize air entrapment and promote polymer setting.

#### 4.6.3. Encapsulation of Bovine Colostrum

Prior to the incorporation of the bovine colostrum, each complex coacervation preparation was heated to 50 °C. Subsequently, the bovine colostrum was added to the mixture and stirred until homogeneous. Following that, a 10% maltodextrin solution was introduced to the suspension and mixed thoroughly. The resulting suspension was then heated at 70 °C for 2 min [94]. The resulting mixtures were homogenized at a speed of 11,000 rpm using an IKA Ultra-Turrax T25 (Works Inc., Wilmington, NC, USA) for a duration of 5 min [94]. Subsequently, an aliquot of the resulting mixture was collected for the determination of entrapment efficiency and loading capacity. The remainder of the resulting mixture was frozen and subjected to a freeze-drying process for a duration of 24 h at −70 °C, under a pressure range of 3 to 15 Pas using an EYELA FDU-2110 freeze-dryer (Tokyo Rikakikai Co., Ltd., Tokyo, Japan). The resultant powder was stored in a vacuum in a laminated foil bag and kept at room temperature until further analysis [95].

### 4.7. Characterization of EBC

#### 4.7.1. Physical Characteristics

The physical characteristics of EBC were visually and organoleptically assessed in terms of external appearance and odor. The color of each EBC was determined using a 3nh NH310 portable colorimeter (Shenzhen Threenh Technology Co., Ltd., Shenzhen, China). Five stochastic points of each EBC were evaluated. The color parameters, including L*, a*, and b*, were recorded, where L* represents the variation from black (0) to white (100), a* represents the variation from green (−) to red (+), and b* represents the variation from blue (−) to yellow (+). The measurements were performed with three replicates at 25 °C.

#### 4.7.2. Microstructural Analysis by Scanning Electron Microscopy (SEM)

The surface morphologies of freeze-dried bovine colostrum and each EBC system were determined using SEM (Nova Nano SEM 230, FEI, Hillsborough, OR, USA) at magnifications indicated in the Results section.

#### 4.7.3. Yield

The yield of freeze-dried bovine colostrum and each EBC system was calculated using the following equation:

Yield (% *w*/*w*) = (A/B) × 100, (1)

where A represents the weight of freeze-dried bovine colostrum powder or EBC powder after freeze-drying and B represents the initial weight of the combination of bovine colostrum, biopolymer, and maltodextrin.

#### 4.7.4. Water Activity

The water activity of each EBC, which is defined as the ratio of the equilibrium vapor pressure of the sample to the equilibrium vapor pressure of pure water at the same temperature [96], was determined using a water activity meter (Novasina LabMaster-aw NEO, Novasina AG, Zurich, Switzerland). In brief, approximately 2 g of the dried EBC powder was weighed in a dedicated container, manually dispersed into a thin layer, and then subjected to the water activity meter. The measurements were performed with three replicates at 25 °C.

#### 4.7.5. Moisture Content

The moisture content of each EBC was determined using a moisture analyzer (ML-50, A&D Company, Limited, Tokyo, Japan). In brief, approximately 2 g of the dried EBC powder was weighed in a dedicated container, manually dispersed into a thin layer, and then subjected to the moisture analyzer, which was set to a drying temperature of 120 °C. The measurements were performed with three replicates at 25 °C.

#### 4.7.6. Water Solubility

The solubility of each EBC in water was determined using a method modified from Jia et al.’s (2020) [97]. In brief, each EBC was accurately weighed and placed into a centrifuge tube. An accurate volume of water was then added, and the combination was mixed using a vortex mixer for a duration of 30 s and stirred at 700 rpm for 30 min. Subsequently, the mixture was centrifuged at 4200 rpm for 30 min using a Centrifuge 5804 R (Eppendorf SE, Hamburg, Germany). The supernatant was then collected and oven-dried at 105 °C overnight. The solubility was calculated based on the weight difference using the following equation:

Water solubility (g/L) = A/B,(2)

where A represents the weight of the residue from drying the supernatant and B represents the volume of distilled water. The measurements were performed with three replicates at 25 °C.

#### 4.7.7. Hygroscopicity

The hygroscopicity of each EBC was determined according to the method described by Li et al. (2022) [98]. In brief, 1.0 g of each EBC was placed in a Petri dish and positioned in a chamber containing a saturated NaCl solution to achieve a relative humidity (RH) of 75%. Hygroscopicity ability was presented as a percentage of the water absorption of the dried powder after one week of storage [99], which was calculated using the following equation:

Hygroscopicity (% *w*/*w*) = (A − B)/B × 100, (3)

where A represents the final weight of the EBC and B represents the initial weight of the EBC. Measurements were performed with three replicates at 25 °C.

#### 4.7.8. Stability Test

The freeze-dried bovine colostrum and its encapsulations were evaluated for both physical and chemical stability through repetitive temperature fluctuations in heating–cooling cycles at 45 °C for 24 h and 4 °C for 24 h (1 cycle). This process was repeated for a total of eight cycles. After the stability test, all samples were subjected to characterization of their physical characteristics, color, water activity, moisture content, water solubility, and hygroscopicity [100,101]. Additionally, the bovine colostrum contents were determined using a bicinchoninic acid (BCA) assay, which is a widely used colorimetric method to quantify protein concentrations [102]. In brief, the sample was dissolved in DI water at a concentration of 10 mg/mL. Subsequently, 25 µL of the sample solution was mixed with 200 µL of the BCA working reagent and incubated at 37 °C for 30 min. Finally, the absorbance of the resulting mixtures was at 562 nm. Analyses were performed with three replicates.

### 4.8. Determination of Entrapment Efficiency and Loading Capacity of EBC

#### 4.8.1. Validation of Entrapment Efficiency and Loading Capacity Determination Method

To verify the accuracy of the methods for determining entrapment efficiency and loading capacity, the EBC and native bovine colostrum were evaluated for their morphology by optical microscopy (Meiji Techno Co., Ltd., Saitama, Japan) and TEM (Jeol Ltd., Tokyo, Japan) in comparison with their supernatant after centrifugation at 4000 rpm for 20 min. In addition, their total protein contents were also evaluated using the BCA assay. The analyses were performed with three replicates.

#### 4.8.2. Determination of Entrapment Efficiency and Loading Capacity of EBC by an Indirect Method

The encapsulation efficiency and loading capacity of each EBC were determined following the method by Kocić et al. (2017) with some modifications [103,104,105]. In brief, each EBC was centrifuged at 4000 rpm for 20 min. Subsequently, the supernatant was collected and analyzed for the bovine colostrum content using the BCA assay. The analyses were performed with three replicates. The encapsulation efficiency (% *w*/*w*) and the loading capacity (% *w*/*w*) were calculated using the following equations:

Encapsulation efficiency (% *w*/*w*) = (A − B)/A × 100, (4)

Loading capacity (% *w*/*w*) = (A − B)/C × 100, (5)

where A represents the total protein content of bovine colostrum, B represents the total protein content of supernatant from the experiments, and C represents the total weight of the encapsulation. The analyses were performed with three replicates at 25 °C.

### 4.9. Development of Blank Powders for Facial Masks

#### 4.9.1. Preparation of Blank Powders for Facial Masks

A blank powder for a facial mask was developed to be freshly mixed with water before application. Various types of blank powder for facial masks were developed in the current study, including conventional clay masks (Table 15), peel-off gel masks (Table 16), and sleeping gel masks (Table 17).

#### 4.9.2. Characterizations of Blank Powder for Facial Mask

Each blank powder for the facial mask was characterized based on its external appearance through visual and organoleptic assessments, along with measurements of color parameters (L*, a*, b*), water activity, moisture content, water solubility, and hygroscopicity.

#### 4.9.3. Determining the Optimal Water-to-Mask Powder Ratio

Since the powder for the facial mask is intended to be mixed with water before application to the face, the optimum powder-to-water ratio needed to be determined. In brief, the blank powder for the facial mask was mixed with various ratios of water. Powder-to-water ratios of 1:1, 1:2, and 1:3 were used for the preparation of conventional clay masks. Powder-to-water ratios of 1:1, 1:2, 1:3, and 1:4 were used for the preparation of facial peel-off gel masks, whilst the powder-to-water ratios of 1:5, 1:10, and 1:15 were used for the preparation of the facial sleeping gel mask. The resulting formulations were subsequently characterized in terms of physical characteristics and pH. Prior to the pH measurement, each formulation was mixed with water in specific ratios, i.e., 1:1, 1:2, and 1:3 for the conventional facial clay mask; 1:1, 1:2, 1:3, and 1:4 for the peel-off mask; and 1:5, 1:10, and 1:15 for the sleeping mask. Subsequently, the pH was measured using a pH meter (C5010, Consort BVBA, Turnhout, Belgium). In the case of conventional clay masks and facial peel-off gel masks, each formulation was tested for drying time by spreading 1 g of freshly prepared mask into a thin, uniform layer about 1 mm thick on a glass plate and maintaining it at 31.0 ± 2.0 °C to simulate human skin temperature. The formulations were monitored until the drying process was completed, as indicated by reaching a constant weight [106].

### 4.10. Development of Powders for Facial Masks Containing EBC

The most suitable formulation of each type of blank powder for the facial mask was selected for the incorporation of EBC. Various concentrations of EBC were incorporated into the blank powder for facial masks, with 50% *w*/*w* used for conventional clay masks and facial sleeping gel masks, and 20% *w*/*w* for facial peel-off gel masks. Subsequently, the formulation’s external appearance was characterized through visual and organoleptic assessments, along with measurements of water activity, moisture content, water solubility, hygroscopicity, and stability. Their stability was tested through eight cycles of temperature fluctuations, alternating between 45 °C for 24 h and 4 °C for 24 h. In addition, the optimum amount of water was applied to each powder containing EBC, and the formulation’s external appearance, pH, and drying time were characterized.

### 4.11. Statistical Analysis

The data are expressed as mean ± SD and were statistically analyzed using IBM SPSS Statistics for Windows Version 20.0. The resulting statistical significance was assessed using analysis of variance (ANOVA) followed by Tukey’s post hoc test or unpaired *t*-test. The level of significant difference was set at * *p* < 0.05.

## Figures and Tables

**Table 1:** The utilities of bovine colostrum in pharmaceutical and cosmetic areas.

Area of Utilizations	Utilities of Bovine Colostrum
Pharmaceutical	Used as an antibacterial agent until the development of antibiotics [11] Treatment of diarrhea due to bacterial, viral, and protozoal pathogens [12] Neutralization of lipopolysaccharide (enterogenic translocation) [12] Treatment of gut-barrier diseases and inflammatory bowel diseases [13] Functional foods and supplements for athletes [14,15] Topical treatment option for the treatment of psoriatic plaque [16]
Cosmetic	Promotes migration of the human keratinocytes and re-epithelialization at the wound site [17] Upregulates skin cell proliferation [17] Heals and repairs damaged and aged skin cells [18] Decreases melanin production by the regulation of the cyclic adenosine monophosphate (cAMP) signaling pathway [19]

**Table 2:** Nutritional compositions of bovine colostrum.

Nutritional Compositions	Amount (% *w*/*w*)
Fat	4.80 ± 0.99
Solids-not-fat	13.14 ± 0.22
Protein	9.00 ± 0.20
Lactose	3.35 ± 0.02
Mineral and vitamin	0.80 ± 0.01

**Table 3:** Amino acid composition of freeze-dried bovine colostrum.

Amino Acids	Amount (% *w*/*w*)
Glutamic acid	3.60
Leucine	1.75
Proline	1.50
Aspartic acid	1.48
Lysine	1.46
Valine	1.24
Serine	1.11
Threonine	0.89
Phenylalanine	0.86
Tyrosine	0.86
Isoleucine	0.86
Arginine	0.69
Alanine	0.65
Histidine	0.55
Glycine	0.43
Methionine	0.41
Tryptophan	0.26
Cystine	<0.20
Hydroxylysine	ND
Hydroxyproline	ND

NOTE: The limit of detection was 0.20% *w*/*w*; ND = not detected.

**Table 4:** Color measurement of the freeze-dried bovine colostrum and encapsulated bovine colostrum (EBC) prepared by complex coacervation using various ratios of gelatin and gum Arabic.

Gelatin and Gum Arabic Ratio	L*	a*	b*
0:0	93.27 ± 0.14	1.09 ± 0.09 ^a^	18.29 ± 0.23 ^a^
1:0	93.70 ± 0.90	−0.01 ± 0.05 ^c^	9.47 ± 0.16 ^b,c^
2:1	93.64 ± 0.95	0.33 ± 0.09 ^b^	10.74 ± 0.33 ^b^
1:1	93.92 ± 0.74	0.43 ± 0.02 ^b^	10.92 ± 0.11 ^b^
1:2	94.51 ± 0.74	0.49 ± 0.06 ^b^	9.79 ± 0.36 ^c^
0:1	94.21 ± 0.11	0.35 ± 0.07 ^b^	9.02 ± 0.24 ^d^

NOTE: L* represents the variation from black (0) to white (100), a* represents the variation from green (−) to red (+), and b* represents the variation from blue (−) to yellow (+). Values are expressed as the mean ± SD of triplicates. Lowercase letters (a, b, c, and d) in each column indicate statistically significant differences among samples, as determined by one-way ANOVA followed by Tukey’s post hoc test (*p* < 0.05).

**Table 5:** Yields, water activity, moisture content, water solubility, hygroscopicity, and protein content of the freeze-dried bovine colostrum and encapsulated bovine colostrum (EBC) prepared by complex coacervation method using various ratios of gelatine and gum Arabic.

Gelatin and Gum Arabic Ratio	Yield (% *w*/*w*)	Water Activity	Moisture Content (% *w*/*w*)	Water Solubility (g/L)	Hygroscopicity (% *w*/*w*)
0:0	14.16 ± 0.21	0.15 ± 0.00 ^a^	4.02 ± 0.49	43.58 ± 0.74 ^d^	10.53 ± 1.25
1:0	15.86 ± 1.82	0.13 ± 0.01 ^b^	4.25 ± 0.15	62.36 ± 0.79 ^a^	11.88 ± 1.37
2:1	16.40 ± 1.80	0.12 ± 0.00 ^b^	4.02 ± 0.04	59.46 ± 0.68 ^c^	10.66 ± 0.73
1:1	17.28 ± 2.91	0.11 ± 0.00 ^b^	3.88 ± 0.13	60.11 ± 0.73 ^bc^	10.56 ± 1.02
1:2	16.47 ± 1.66	0.12 ± 0.00 ^b^	4.23 ± 0.36	58.60 ± 0.56 ^c^	11.77 ± 1.23
0:1	15.91 ± 1.69	0.13 ± 0.01 ^b^	4.22 ± 0.17	60.11 ± 0.85 ^ab^	11.98 ± 0.24

NOTE: Values are expressed as the mean ± standard deviation, with each measurement performed in triplicate. Lowercase letters (a, b, c, and d) in each column indicate statistically significant differences among samples, as determined by one-way ANOVA followed by Tukey’s post hoc test (*p* < 0.05).

**Table 6:** Total protein content of bovine colostrum and its encapsulation, along with the supernatants after centrifugation at 4000 rpm for 20 min.

Samples	Total Protein Content (mg/mL)
Bovine colostrum	9.3 ± 0.1 ^b^
Supernatant of centrifuged bovine colostrum	8.4 ± 0.2 ^c^
Encapsulated bovine colostrum dispersion	9.8 ± 0.0 ^a^
Supernatant of centrifuged encapsulated bovine colostrum	4.6 ± 0.1 ^d^

NOTE: Values are expressed as the mean ± standard deviation, with each measurement performed in triplicate. Lowercase letters (a, b, c, and d) in each column indicate statistically significant differences, as determined by one-way ANOVA followed by Tukey’s post hoc test (*p* < 0.05).

**Table 7:** Entrapment efficiency and loading capacity of bovine colostrum in the encapsulation systems prepared by complex coacervation method using various ration of gelatin and gum Arabic.

Gelatin and Gum Arabic Ratio	Entrapment Efficiency (% *w*/*w*)	Loading Capacity (% *w*/*w*)
0:0	10.0 ± 1.1 ^d^	6.5 ± 0.6 ^d^
1:0	69.0 ± 3.1 ^a^	70.0 ± 3.1 ^a^
2:1	66.6 ± 3.3 ^a^	67.6 ± 3.4 ^a^
1:1	53.9 ± 4.2 ^b^	54.7 ± 4.2 ^b^
1:2	41.0 ± 0.8 ^c^	41.6 ± 0.8 ^c^
0:1	34.1 ± 1.6 ^c^	34.6 ± 1.7 ^c^

NOTE: Values are expressed as the mean ± standard deviation, with each measurement performed in triplicate. Lowercase letters (a, b, c, and d) in each column indicate statistically significant differences, as determined by one-way ANOVA followed by Tukey’s post hoc test (*p* < 0.05).

**Table 8:** External appearance and characteristics of blank powder for conventional facial clay mask.

Characteristics	Formulation		
	C1	C2	C3
External appearance	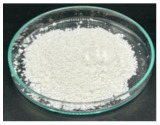	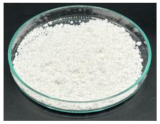	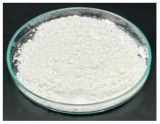
	White fine powder	White fine powder	White fine powder
Color parameters			
L*	95.22 ± 0.53	95.23 ± 0.33	94.84 ± 0.12
a*	0.35 ± 0.08 ^a^	0.29 ± 0.05 ^a^	0.14 ± 0.02 ^b^
b*	4.27 ± 0.30 ^a^	4.16 ± 0.13 ^a^	3.48 ± 0.19 ^b^
Water activity	0.45 ± 0.00 ^a^	0.46 ± 0.00 ^a^	0.41 ± 0.00 ^b^
Moisture content (% *w*/*w*)	0.88 ± 0.15	0.88 ± 0.12	0.84 ± 0.11
Water solubility (g/L)	1.57 ± 0.06 ^b^	1.64 ± 0.02 ^b^	1.74 ± 0.03 ^a^
Hygroscopicity (% *w*/*w*)	0.26 ± 0.03	0.23 ± 0.03	0.25 ± 0.14

NOTE: Values are expressed as the mean ± standard deviation, with each measurement performed in triplicate. Lowercase letters (a and b) in each column indicate statistically significant differences, as determined by one-way ANOVA followed by Tukey’s post hoc test (*p* < 0.05).

**Table 9:** External appearance and characteristics of blank powder for conventional facial clay mask formulation C3 (see Table 8) after mixing with water at various ratios.

Characteristics	Powder-To-Water Ratio for Facial Masks		
	1:1	1:2	1:3
External appearance	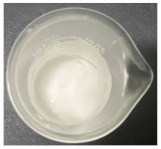	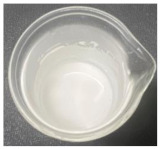	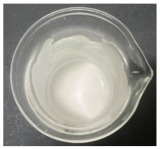
	White paste	White paste	White paste
pH	6.75 ± 0.01	6.75 ± 0.01	6.75 ± 0.01
Drying time (min)	7.67 ± 0.58 ^b^	8.00 ± 1.00 ^b^	12.67 ± 1.15 ^a^

NOTE: Values are expressed as the mean ± standard deviation, with each measurement performed in triplicate. Lowercase letters (a and b) in each column indicate statistically significant differences, as determined by one-way ANOVA followed by Tukey’s post hoc test (*p* < 0.05).

**Table 10:** External appearance and characteristics of blank powder for facial peel-off gel mask.

Characteristics	Formulation		
	P1	P2	P3
External appearance	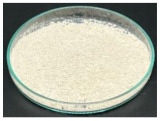	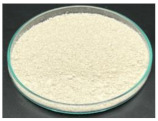	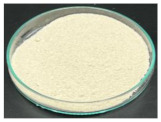
	Light-yellow powder	Light-yellow powder	Light-yellow powder
Color parameters			
L*	95.95 ± 0.20 ^a^	94.97 ± 0.11 ^b^	93.94 ± 0.12 ^c^
a*	0.40 ± 0.62 ^c^	0.54 ± 0.02 ^b^	0.63 ± 0.02 ^a^
b*	4.52 ± 0.18 ^a^	5.09 ± 0.10 ^b^	5.80 ± 0.05 ^c^
Water activity	0.36 ± 0.00	0.38 ± 0.04	0.39 ± 0.00
Moisture content (% *w*/*w*)	4.71 ± 0.04 ^a^	4.58 ± 0.07 ^a^	4.18 ± 0.31 ^b^
Water solubility (g/L)	0.02 ± 0.00 ^c^	0.05 ± 0.01 ^b^	0.07 ± 0.02 ^a^
Hygroscopicity (% *w*/*w*)	0.71 ± 0.11	0.79 ± 0.02	0.74 ± 0.06

NOTE: Values are expressed as the mean ± standard deviation, with each measurement performed in triplicate. Lowercase letters (a, b and c) in each column indicate statistically significant differences, as determined by one-way ANOVA followed by Tukey’s post hoc test (*p* < 0.05).

**Table 11:** External appearance and characteristics of blank powder for facial peel-off gel mask formulation P3 after mixing with water at various ratios.

Characteristics	Powder-To-Water Ratio for Facial Masks			
	1:1	1:2	1:3	1:4
External appearance	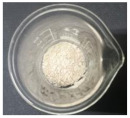	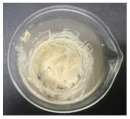	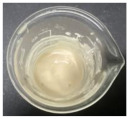	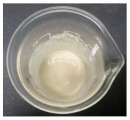
	Light-yellow powder	Light-yellow, opaque paste	Light-yellow, opaque gel	Light-yellow, opaque liquid
pH	NA	6.00 ± 0.00 ^b^	6.50 ± 0.00 ^a^	6.50 ± 0.00 ^a^
Drying time (min)	NA	35.00 ± 6.24 ^b^	44.33 ± 0.58 ^a^	43.67 ± 1.53 ^a^

NOTE: NA: not available. Values are expressed as the mean ± standard deviation, with each measurement performed in triplicate. Lowercase letters (a and b) in each column indicate statistically significant differences, as determined by one-way ANOVA followed by Tukey’s post hoc test (*p* < 0.05).

**Table 12:** External appearance and characteristics of blank powder for facial sleeping gel masks.

Characteristics	Formulation		
	S1	S2	S3
External appearance	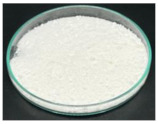	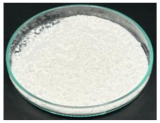	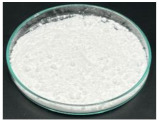
	White powder	White powder	White powder
Color parameters			
L*	96.15 ± 0.37 ^b^	96.31 ± 0.20 ^a,b^	96.76 ± 0.24 ^a^
a*	0.44 ± 0.06 ^b^	0.50 ± 0.03 ^a,b^	0.59 ± 0.10 ^a^
b*	3.63 ± 0.19 ^a^	3.39 ± 0.11 ^a,b^	3.26 ± 0.04 ^b^
Water activity	0.25 ± 0.00 ^a^	0.23 ± 0.00 ^b^	0.22 ± 0.00 ^b^
Moisture content (% *w*/*w*)	3.65 ± 0.55 ^b^	4.31 ± 0.21 ^b^	5.32 ± 0.22 ^a^
Water solubility (g/L)	NA	NA	NA
Hygroscopicity (% *w*/*w*)	8.74 ± 0.80	9.14 ± 0.19	9.07 ± 0.49

NOTE: NA: not available. Values are expressed as the mean ± standard deviation, with each measurement performed in triplicate. Lowercase letters (a and b) in each column indicate statistically significant differences, as determined by one-way ANOVA followed by Tukey’s post hoc test (*p* < 0.05).

**Table 13:** External appearance and characteristics of blank powder for facial sleeping gel mask formulation S2 after mixing with water at various ratios.

Characteristics	Powder-To-Water Ratio for Facial Masks		
	1:5	1:10	1:15
External appearance	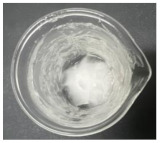	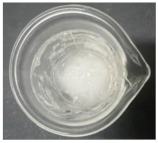	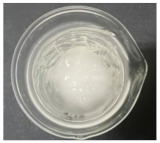
	White, opaque gel	White, opaque gel	White, opaque gel
pH	5.50 ± 0.00 ^b^	5.75 ± 0.00 ^a^	5.70 ± 0.09 ^a^

NOTE: Values are expressed as the mean ± standard deviation, with each measurement performed in triplicate. Lowercase letters (a and b) in each column indicate statistically significant differences, as determined by one-way ANOVA followed by Tukey’s post hoc test (*p* < 0.05).

**Table 14:** External appearance and characteristics of powder for facial mask containing encapsulated bovine colostrum (EBC) and formulations after mixing with water.

Characteristics	Formulation					
	C3		P3		S2	
Concentration of EBC (% *w*/*w*)	0	50	0	20	0	50
External appearance of powder for facial mask containing EBC	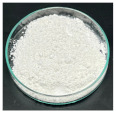	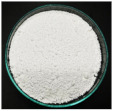	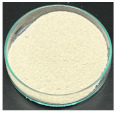	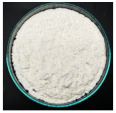	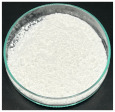	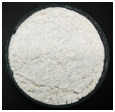
	White powder	White powder	Light-yellow powder	White powder	White powder	White powder
Color parameters						
L*	94.84 ± 0.12	94.63 ± 0.19	93.94 ± 0.12	93.85 ± 0.13	96.31 ± 0.20	95.69 ± 0.22
a*	0.14 ± 0.02	0.53 ± 0.02 *	0.63 ± 0.02	0.71 ± 0.02 *	0.50 ± 0.03	0.39 ± 0.04 *
b*	3.48 ± 0.19	8.20 ± 0.09 *	5.80 ± 0.05	5.94 ± 0.03 *	3.39 ± 0.11	8.35 ± 0.12 *
Water activity	0.41 ± 0.00	0.44 ± 0.18	0.39 ± 0.00	0.24 ± 0.02 *	0.16 ± 0.00	0.45 ± 0.02 *
Moisture content (% *w*/*w*)	0.84 ± 0.11	2.39 ± 0.44 *	4.18 ± 0.31	3.85 ± 0.64	4.31 ± 0.21	4.23 ± 0.28
Water solubility (g/L)	1.74 ± 0.03	11.40 ± 0.31 *	0.07 ± 0.02	11.54 ± 0.30 *	NA	NA
Hygroscopicity (% *w*/*w*)	0.25 ± 0.14	3.29 ± 0.04 *	0.74 ± 0.06	2.80 ± 0.63 *	9.14 ± 0.19	10.58 ± 0.34
Powder-to-water ratio for facial masks	1:1	1:1	1:3	1:3	1:10	1:10
External appearance after mixing with water	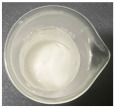	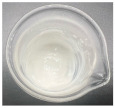	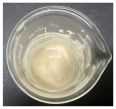	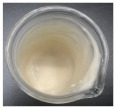	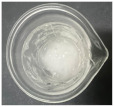	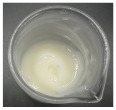
	White paste	White paste	Light-yellow, opaque gel	Light-orange, opaque gel	White, opaque gel	Light-yellow, opaque gel
pH	6.75 ± 0.00	6.00 ± 0.00 *	6.50 ± 0.00	6.08 ± 0.58 *	5.75 ± 0.00	5.75 ± 0.03
Drying time (min)	7.67 ± 0.58	9.67 ± 0.58	44.33 ± 0.58	25.00 ± 1.73 *	ND	ND

NOTE: NA: not available; ND: not determined. Values are expressed as the mean ± standard deviation, with each measurement performed in triplicate. Asterisk (*) indicates statistically significant differences between the formulation with and without encapsulated bovine colostrum (EBC), as determined by paired t-test (*p* < 0.05).

**Table 15:** Ingredients in blank conventional clay mask formulations.

Ingredients	Concentration (% *w*/*w*)		
	C1	C2	C3
Talc	40	30	20
Kaolin	45	55	65
Titanium dioxide	6	6	6
Magnesium aluminum silicate	5.5	5.5	5.5
Silica	3	3	3
Allantoin	0.3	0.3	0.3
Imidazolidinyl urea	0.2	0.2	0.2

**Table 16:** Ingredients in blank peel-off gel mask formulations.

Ingredients	Concentration (% *w*/*w*)		
	P1	P2	P3
Sodium alginate	12.5	12.5	12.5
Calcium sulfate	8	8	8
Diatomaceous earth	55	65	75
Talc	22	12	2
Tetrasodium pyrophosphate	2.45	2.45	2.45
Sodium metabisulfite	0.025	0.025	0.025
Sodium sulfite	0.025	0.025	0.025

**Table 17:** Ingredients in blank sleeping gel mask formulations.

Ingredients	Concentration (% *w*/*w*)		
	S1	S2	S3
HK-AVC03	20	40	60
Niacinamide	62	42	22
Allantoin	8	8	8
*Aloe vera* powder	2	2	2
Sodium hyaluronate	2	2	2
Disodium EDTA	2	2	2
Imidazolidinyl urea	4	4	4

## Data Availability

The original contributions presented in this study are included in the article. Further inquiries can be directed to the corresponding author.
